# The Destruction of the Poor

**Published:** 1887-09-17

**Authors:** 


### "THE DESTRUCTION OF THE POOR."

"The destruction of the poor is their poverty," said the wise man. He probably meant by "poverty" the want of money or material resources. But there are kinds of poverty which a high civilisation brings to light that are ten times worse than the mere want of money or any other material equivalent whatsoever. There is the poverty of courage and resolution; the poverty of sense and will; the poverty of self-respect and manhood. The Exeter Theatre has just been burnt to the ground, and a hundred and fifty human beings have been burnt or destroyed with it. Those hundred and fifty were mostly poor. The people who meet their deaths at theatres, in railway accidents, and other public calamities, generally are poor. It is only on the sea, when some terrible wreck or "ship on fire" is witnessed, that the rich and the poor are involved in a common destruction.

There were many people in the Exeter Theatre when the fire broke out who belonged to the wealthier classes. All, or almost all, of these filed out steadily and safely, and reached their homes in peace whilst their fellows were being crushed, suffocated, and burnt to death. Now that the first feelings of horror and dismay are past, we need not waste our energies and sympathies in mere expressions of natural but ineffective pity. Rather let us take the whole question of such dangers into consideration, and see if such calamities ought not to be rendered for ever impossible. Why were those hundreds of poor people packed away into what was bound to be a place of terrible danger, if not of death, in the event of fire? Does anyone mean to say that it is not possible to construct a public building like a theatre in such a manner as that if a fire breaks out escape shall be safe and easy? Of course it is possible; and if all theatre-goers consisted of rich men and women whose lives are valued by themselves and other people, there would never be another calamity like that at Exeter so long as the world stands. People are stuffed away into top galleries at the end of long, narrow, and dangerous passages, because they cannot pay for more expensive places; but also because they have not the foresight and energy of character to refuse to be packed away into such regions. At bottom it is a want of sense and strength of mind which allows such things to happen. If playgoers were resolved either to enjoy the play in safety, or not to enjoy it at all, every theatre in the civilised world would soon be as safe in the topmost gallery as it is now in the boxes and stalls.

In the first instance, the playgoers who enter dangerous galleries are themselves to be blamed; and it may be said with certainty that when this calamity is forgotten, as it will be in a month, they will continue to run just as great risks as ever, unless they take the matter into their own hands. Do we, then, exonerate the builders and managers of theatres? By no means. They will have their reckoning-day in due time. But builders and managers do that which all men do: they consult their own interests first of all; and their interests are to lay out as little money as possible in the building of the theatre. They are concerned with profits and dividends, and it is perfectly natural and inevitable that they should be so concerned. They will not—and it is not in human nature that they should—think more of the interests of any other class than they do of their own. To them, therefore, it is utterly futile to look for any change except on compulsion; and to blame them for this would be to take up a foolish and impracticable, if not a pharisaical position.

But the necessary changes ought now to be made, and made without any kind of doubt, so that theatre-going people may no longer be compelled to take their pleasure at the mouth of a volcano. Who is to make these changes? Can the municipalities in provincial towns or Parliament in the metropolis be expected to take the matter up? Of their own motion—no! If sufficient pressure be brought to bear upon them—yes! Who is to bring the pressure to bear? Will the rich do it? They will not. Why should they? No doubt it would be generous and humane if they did; but they will not. And we repeat, not in an unkindly but in a practical spirit, instructed by a knowledge of human nature, why should they? If the necessary changes are to be made, it is only the poorer class of playgoers who can supply the motive power. If they have sense and energy enough, they can move the arms, not only of managers, but of municipalities and of Parliament itself. They must combine. Alone they are powerless and contemptible: in combination they would be mighty and irresistible. It may be said that these counsels are unpractical. They are unpractical if they be addressed to thoughtless, weak-minded, and unpractical people. Are the poorer classes of play-goers correctly described by these terms? Have they not got mind enough to see that they must rouse themselves on their own behalf if any good is to be done; or, having mind enough to see, have they not got sufficient force of character to initiate a movement in the direction of safety? What kind of a confession is that to make? If the result of attending theatres be to convert human beings into such poor creatures as that, the sooner all theatres are closed by Act of Parliament the better.

Innumerable suggestions are made in the daily papers for making theatres as safe as private houses. The suggestions are good enough in many instances, but quite superfluous. It is not that there is any difficulty at all in making such places safe. To suppose that is to gratuitously insult the whole profession of architects and practical builders. There are undoubtedly half-a-dozen possible plans by which any theatre can be rendered a house of safety provided sufficient money be spent in the operation. It is, in a sense, a question of money, but, as our whole contention goes to show, still more of some way of compelling the money to be spent. Parliament can with ease and promptitude pass such a measure as will ensure a reasonable degree of safety to all playgoers, by compulsory structural changes, which changes may be made in six months. Parliament is the lever. Where are the fulcrum and the power? Public opinion is so roused by the Exeter calamity that it may be considered a fulcrum lying ready to hand. If playgoers would but resolve to rouse themselves to action, instead of wringing their hands in weak and helpless despair, the heart-breaking loss of life at Exeter would not have been in vain. It is much to be feared, however, that all those who have not personally suffered will speedily forget all about it. But who can tell which may be the next theatre to emphasise the lesson of foresight and self-preservation? The mere prospect of another disaster of the kind appals the stoutest heart. And in spite of logic and political economy, and the conviction that the proper persons to initiate measures of safety are those who are most likely to suffer by the danger, we cannot but hope that Parliament may of its own motion pass such measures as shall render these terrible calamities at once and for ever impossible.

